# Diabetes mellitus in peripheral artery disease: Beyond a risk factor

**DOI:** 10.3389/fcvm.2023.1148040

**Published:** 2023-04-17

**Authors:** Madhu V. Singh, Ayotunde O. Dokun

**Affiliations:** ^1^ Division of Endocrinology and Metabolism, Department of Internal Medicine, Carver College of Medicine, University of Iowa, Iowa City, IA, United States; ^2^ Fraternal Order of Eagles Diabetes Research Centre, Carver College of Medicine, University of Iowa, Iowa City, IA, United States

**Keywords:** peripheral artery disease, diabetes, ischemia, vascular, gene expression, microRNA, angiogenesis

## Abstract

Peripheral artery disease (PAD) is one of the major cardiovascular diseases that afflicts a large population worldwide. PAD results from occlusion of the peripheral arteries of the lower extremities. Although diabetes is a major risk factor for developing PAD, coexistence of PAD and diabetes poses significantly greater risk of developing critical limb threatening ischemia (CLTI) with poor prognosis for limb amputation and high mortality. Despite the prevalence of PAD, there are no effective therapeutic interventions as the molecular mechanism of how diabetes worsens PAD is not understood. With increasing cases of diabetes worldwide, the risk of complications in PAD have greatly increased. PAD and diabetes affect a complex web of multiple cellular, biochemical and molecular pathways. Therefore, it is important to understand the molecular components that can be targeted for therapeutic purposes. In this review, we describe some major developments in enhancing the understanding of the interactions of PAD and diabetes. We also provide results from our laboratory in this context.

## Introduction

Peripheral artery disease (PAD) is the atherosclerotic occlusion of the peripheral arteries of the lower extremities. It is the third largest cardiovascular disease that afflicts more than 200 million people worldwide (1). A large number of cases go undiagnosed because often the patients have no complaint, and the symptoms and signs of the disease may appear only at an advanced stage of the disease. These symptoms primarily originate from diminished blood flow to the muscular tissue and range from intermittent claudication or pain with ambulation. Individuals may also present with rest pain, non-healing wounds, ulceration, and gangrene which are hallmarks of the more severe presentation of the disease termed critical limb threatening ischemia (CLTI). CTLI often leads to amputation and is associated with high mortality rates (2).

PAD has similar risk factors as coronary atherosclerosis, which includes old age, smoking, hypertension and diabetes. Unlike Coronary artery disease (CAD), diabetes and smoking accounts for most of the risk for developing PAD (3). Cardiovascular diseases including PAD are the most common cause of morbidity and mortality among adults with diabetes (4). With the global prevalence of diabetes mellitus rising from 171 million (Year 2000) to 366 million in 2030 (5), the prevalence of PAD and its severity also are expected to increase. However, beyond being a risk factor, the presence of diabetes worsens outcomes in individuals with PAD (6, 7). Among the risk factors for developing CTLI in PAD diabetes has the strongest effect (8). Both clinical data and preclinical studies with experimental models of PAD show that post-ischemic recovery is poor under diabetic conditions (6). Although diabetes in general dramatically increases the risk of developing CTLI and limb amputation (9–13), clinical data show individuals with T1D and PAD are more likely to undergo limb amputations compared to those with T2D (9). Moreover, the cohort with T1D and PAD had amputations at a younger age than those with T2D and PAD. Interestingly, although poor glycemic control has been associated with increased prevalence of PAD (14) and tight glycemic control has been shown to improve microvascular and macrovascular outcomes in type 1 and type 2 DM (15) it is unclear whether glycemic control improves PAD outcomes in individuals with advanced disease such as CTLI. In a mouse model of PAD and DM, glycemic control does improve PAD outcomes (6). These findings are consistent with hyperglycemia in diabetes being a major driver of the worse PAD outcomes in diabetes. However, lack of insulin or duration of hyperglycemia and resulting metabolic consequences of diabetes may also contribute to the observed severity of PAD among individuals with diabetes. To understand the impact of diabetes on the outcomes of PAD, it would be important to first understand the molecular mechanisms and genetic factors driving normal post-ischemic adaptations. Investigation of the impact of diabetes and its associated metabolic abnormalities on the identified molecular mechanisms and genetic factors will likely provide deeper insight into how diabetes exacerbates the outcomes of PAD.

In preclinical studies, several different models of T1D and T2D have been described each with its strengths and weaknesses, modeling different aspects of the human disease. A review of these models is beyond the scope of this review and has been discussed elsewhere (16–18). It is however possible to simulate aspects of PAD in T1D and T2D using well-established mouse models such as Akita mice, (simulates effect of hyperglycemia), high fat diet feeding (HFD, simulated effect of insulin resistance) or a combination of HFD and streptozotocin, STZ, (simulates effect of both insulin resistance and hyperglycemia), in conjunction with hind limb ischemia (HLI) surgery. Although DM is a complex disease with multiple clinical variables, hyperglycemia, insulin resistance, and associated metabolic disorders are common threads that have been studied in preclinical research to gain deeper insight into cardiovascular diseases including PAD. The mouse model, with rich resources of genetic tools, provides opportunities to understand aspects of the molecular, cellular and structural bases of the effects of diabetes on PAD.

## Adaptations associated with PAD

Following vessel occlusion or narrowing in PAD, the poor perfusion leads to reduced oxygen and nutrients to the limb tissues. This results in inability to meet the metabolic demand of the affected tissue and may result in tissue necrosis and loss of function. The main affected tissue in PAD are the lower extremities where the ischemic condition may result in the death of muscle and vascular cells. Nevertheless, following vessel occlusion three major adaptive processes appear to protect the affected limb from limbs loss and this includes: (1) development of collateral blood flow through arteriogenesis, (2) growth of new blood vessels from existing vessels through angiogenesis and (3) skeletal muscle survival and repair processes.

### The impact of diabetes on vascular adaptation in PAD

One of the key early compensatory responses following vessel occlusion is arteriogenesis whereby remodeling of existing arteries results from shear stress. This process is extensively described in a number of reviews (19–22). Vascular remodeling in arteriogenesis involves nitric oxide, recruitment of monocytes, extracellular matrix remodeling, growth factor and chemokine mediated alteration of existing vessels (23–25). Arteriogenesis may be followed by angiogenesis that entails formation of new capillaries in response to hypoxia-related factors and may involve homing of bone marrow derived progenitor cells in the ischemic tissue (26–28). This process involves the action of cytokines and growth factors including vascular endothelial growth factor (VEGF), fibroblast growth factor-1 (aFGF), hepatocyte growth factor (HGF), hypoxia-inducible factor-1a (HIF-1 a), developmental endothelial locus-1 (Del-1), and stroll cell-derived factor-1 (SDF-1) that are pro-angiogenic (29–31).

There is evidence that diabetes impairs arteriogenesis, but the molecular mechanisms involved are poorly understood (32, 33). The metabolic abnormalities associated with diabetes may affect many of the known processes involved in arteriogenesis. One of the first steps in arteriogenesis is the sensing of shear stress and has been shown to be a major trigger for the process. In diabetes shear stress sensing appear to be impaired but the mechanism has not been well described, however a possible role of production of advance glycation end products has been suggested (33–35). Moreover, another essential factor in collateral formation is endothelial nitric oxide synthase (eNOS) and its expression is increased significantly in collateral vessels during arteriogenesis. eNOS mediated NO release has been shown to be impaired in a mouse model of type 1 DM (STZ) and type 2 DM (Lepr^db/db^ mice) (36–38).

**Figure 1:**
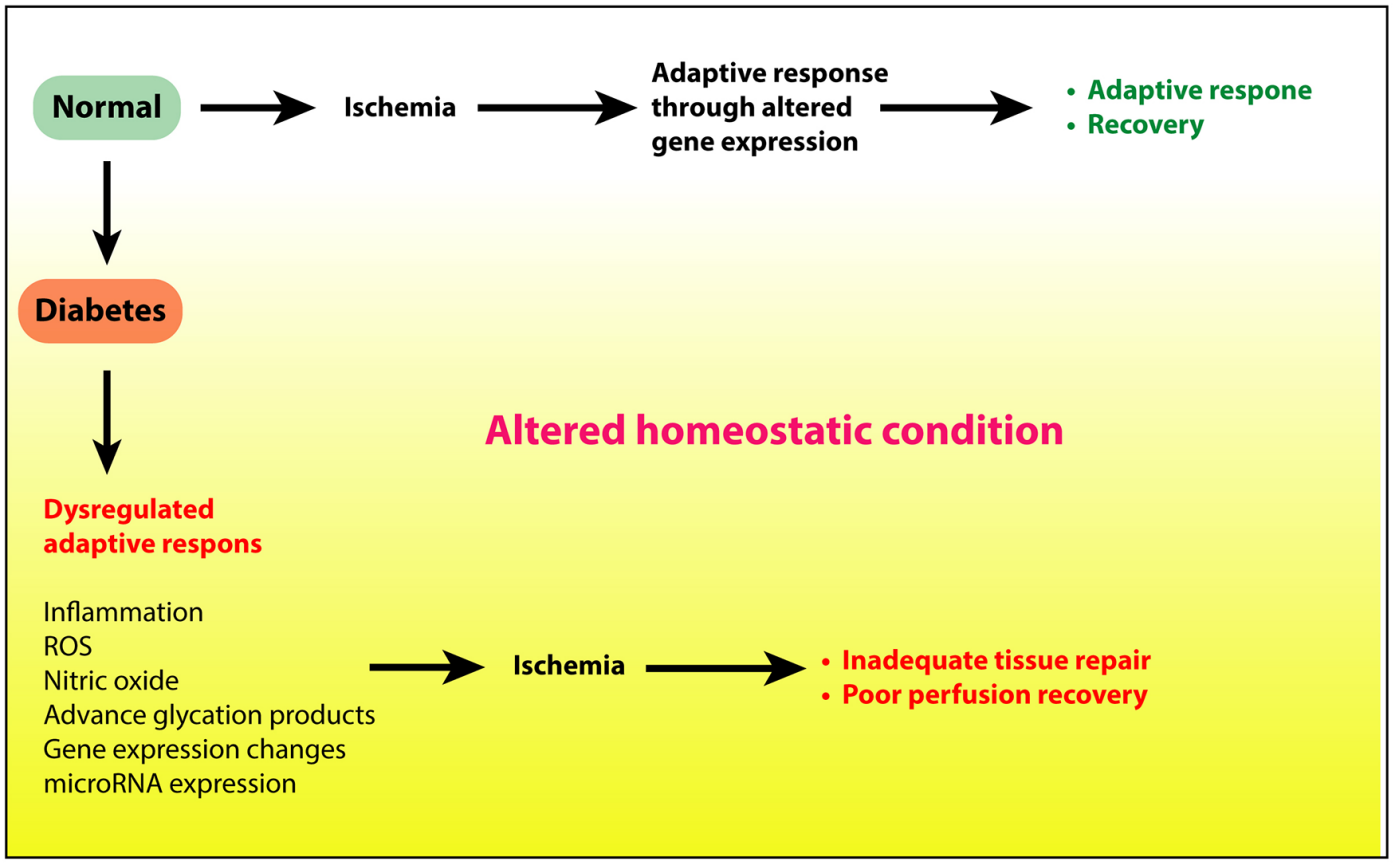
Ischemic injury to limbs under normal conditions elicits adequate adaptive response to restore tissue health and perfusion. However, under diabetic conditions, hyperglycemia, insulin resistance, or a combination of these, alters the tissue homeostatsis through increased inflammation, reactive oxygen species, and other proinflammatory processes. This altered state in turn delays or prevents the adaptive response to repair the tissue.

Diabetes has profound negative effects on ischemic neovascularization of the limb in PAD (33, 39–42). However, the molecular mechanisms of these effects are not well understood. Clearly, the low-grade proinflammatory changes and increased reactive oxygen species (ROS) from hyperglycemia must contribute to this disarray, but additional mechanisms must also be involved. In streptozotocin (STZ)-induced diabetes of NOX2^−/−^ mice, post-HLI vascularization was improved, suggesting a role of oxidative stress in impaired post ischemic angiogenesis in diabetes (43). One of the best investigated aspects of impaired angiogenesis in diabetes is the impact of diabetes on VEGF ligand/receptor expression. The role of VEGF and its receptors as critical signaling pathways in post-ischemic angiogenesis is well established (44–47). In mouse models of type 1 and type 2 diabetes investigators have shown impaired expression of VEGFA protein and mRNA in ischemic mouse hind limbs following experimental induction of PAD (6, 40, 48, 49). Thangarajah et al. showed this was due at least in part to decreased HIF-1alpha activity resulting from impaired HIF-1alpha binding to the coactivator p300 (50). Moreover, Ebrahimian et al. showed normal VEGFA expression was restored in ischemic diabetic limbs by inhibition of ROS formation or by ROS scavenging by the anti-oxidant N-acetyl-l-cysteine (NAC) (43) suggesting impaired VEGF expression was driven by ROS. Studies by our group evaluated the effect of diabetes on expression of VEGF receptors (VEGFR1 and VEGFR2). We found increased expression of soluble VEGFR1 thought to be an inhibitor of VEGFA signaling in ischemic diabetic limbs (40). Additionally, our analysis of VEGFR2 the primary receptor for VEGFA signaling showed decreased expression is ischemic diabetic skeletal muscle. We further demonstrated that this was due to hyperglycemia mediated increased ubiquitination and degradation of the receptor (6). Taken together these studies demonstrate how metabolic changes in diabetes can alter expression and signaling pathways that play important roles in post-ischemic adaptation (Figure 1).

Beyond hyperglycemia, formation of AGEs and ROS activation of protein kinase C (PKC) is another well described finding in diabetic tissues (51, 52). Studies from our lab have shown that persistent hyperglycemia results in impairment of the canonical NF-kB pathway but promotes the non-canonical pathway. This misbalance in NF-kB pathway results from increased PKCb-S664 phosphorylation. Accordingly, in diabetic mice (T1DM, Akita mice), inhibition of PKCb activity by ruboxistaurin improved blood perfusion after hind limb ischemia (51).

The effects of limb ischemia are not limited to the vasculature; the ability of musculature to withstand the ischemic assault and recover from it is also important but remains a less studied area. It is conceivable that without improving the viability and function of the skeletal muscles attempts at revascularization may not suffice for limb preservation in PAD.

### Ischemic limb skeletal muscle effects

Whether the evolution of limb ischemia is chronic or acute, the resulting loss of blood flow adversely affects the perfused tissue. Whereas in chronic ischemia collaterals may be formed as an adaptation to repeated near ischemic episodes, embolic acute ischemia causes more severe damage to the tissue that, if not treated, may lead to infarction and loss of limb. Although several limb muscle groups are affected by ischemia in HLI including the gastrocnemius (GA), the tibialis anterior (TA) and solius (53, 54), owing to its size and volume, the gastrocnemius muscle in mouse model is one of the most studied muscle that is affected by PAD showing both structural changes in the form of denervation, cell death (55), decreased muscle mass, smaller fiber size, fatty deposition, increased number of muscle fibers with central nuclei and biochemical changes (56–58). Centralized nuclei in muscle fibers were originally shown to be in myopathy (59, 60) and also during the repair process in injured muscle. Indeed, a muscle injury entails infiltration of the the tissue by immune cells. However, the size of these cells is usually much smaller than muscle fibers in the cross-sections. Thus, in the absence of a central-nuclear myopathy (CNM), centralized nuclei in muscle fibers are widely considered to be a reflection of the process of repair (61). Moreover, the GA muscle in PAD also show greater mitochondrial impairment, impaired oxidative metabolism, increased reactive oxygen species, and increased fibrosis (58, 62, 63).

Similar to what has been observed in humans, histopathological changes have also been observed in mouse models of PAD (59, 60). The use of mouse models and *ex vivo* studies have provided the tools to better understand the effects of ischemia on the skeletal muscle. The alignment of nuclei in the center of myofibers is a diagnostic feature of centronuclear myopathy, however, in the context of injury, it is a transitionary process of re-localization during repair Accordingly, we also have observed that centralized nuclei in the cross sections of myofibers of ischemic limbs of DM mice persist much longer and are present in higher numbers of fibers than in sections from control non-diabetic ischemic limbs (64).

### Effect of diabetes on ischemic limb skeletal muscle

Reduced blood flow and intermittent ischemia adversely affect the metabolism of the lower limb musculature causing structural damage to the calf muscle area by reducing muscles mass and increased fatty deposits and fibrosis (55). In addition, decreased perfusion leads to impaired mitochondrial function and development of smaller muscle fibers (65). In diabetic patients with PAD, insulin resistance and hyperglycemia may result in adverse metabolic changes in the skeletal muscles causing greater damage and loss of recovery from injury. The metabolic abnormalities in diabetes may also hamper skeletal muscle repair. However, the role of diabetes on skeletal muscle recovery from ischemic injury is relatively less studied. A study in diabetic db/db mice suggests that factors produced in myofibers act upon endothelial cells to modulate their gene expression and function (66). In high fat diet-fed mice with impaired glucose tolerance and HLI surgery, exogenous expression of hepatocyte growth factor and vascular endothelial growth factor 165 improved muscle regeneration by improving innervation and muscle regeneration (67). Studies in our laboratory have shown that following HLI the extent of skeletal muscle injury is more severe in diabetic mice (HFD) compared to non-diabetic and this was associated with a higher rates of limb amputation and poor skeletal muscle function (64). These poorer outcomes may be at least in part due to impaired upregulation of genes important to skeletal muscle adaptation to ischemia (68).

### Effects of diabetes on molecular adaptations in ischemia

In accordance with the ischemic injury, post-ischemic tissue shows marked changes in gene expression. Using microarray-based global gene expression analyses, we have shown that in both type 1 diabetes (Akita mice) and in high fat diet fed type 2 diabetes models there are significantly differences in gene expression in ischemic GA's when compared to expression in non-diabetic mice (69, 70). In both cases, significant changes were observed in the expression of genes involved in metabolic pathways, underlining the role of diabetes on metabolism under ischemic conditions. However, diabetic condition itself alters gene expression (71–73). Diabetic hyperglycemia affects multiple cellular and molecular processes through production of advance glycation end products (AGEs), imbalance in nitric oxide levels, and reactive oxygen species (ROS) through the NF-kB pathways (51, 74–78). Thus, hyperglycemia induces low grade chronic inflammation leading to an altered homeostatic state. In contrast to the non-diabetic state, in diabetes the adaptive response to ischemic injury commences from an altered state, which may not follow the response of the normal state.

### microRNAs in diabetic PAD

MicroRNAs are short RNAs (approximately 22 nucleotides in length) that bind to near complementary seed sequences to their target mRNAs to repress their expression. Although miRNAs primarily target sequences in the 3’UTRs of mRNA, these targets may exist in the coding sequence or in 5’-UTRs. As a large collection of more than 2,000 identified miRNAs in humans, they can potentially regulate one-third of all genes in the human genome. Therefore, it is not surprising that miRNAs would also affect gene expression in PAD. Although a number of studies have looked at the role of miRNAs in PAD as biomarkers or for their regulatory potential for post-ischemic outcomes (79–85), relatively few studies have examined their role in regulating the outcome of PAD in diabetes (64, 86, 87).

T2DM itself can alter the expression of miRNAs. A meta-analysis of 39 case-control studies showed that compared to non-diabetic controls, 494 miRNAs were identified to have differential expression in T2DM patients (88). An interesting feature of studying miRNA is that by targeting a transcription factor they may affect a nodal point and related pathways regulated by the transcription factor (86, 89). In this vein, Feinberg and colleagues (90) identified endothelium-specific miR-375 whose expression was upregulated in the gastrocnemius muscle of non-diabetic mice (db/+) after hind limb ischemia but not in db/db mice. Moreover, miR-375 targets pro-angiogenic pathways through KLF5 transcription factor that is known to play a role in regulation of angiogenesis and skeletal muscle repair. In another study, Caporali et al. showed that expression of miR-503 was upregulated in endothelial cells both *in vitro* in simulated hyperglycemia or ischemia and *in vivo* in ischemic limbs of mice and in PAD patients (89). Inhibition of miR-503 in ischemic limbs of mice improved angiogenesis and blood flow recovery. Mechanistically, miR-503 expression is induced by NF-kB transcription factor and in turn regulates interaction between endothelial cells and pericytes to affect angiogenesis (86).

In our own studies, we have demonstrated that miR-29a influences the recovery from hind limb ischemia in diabetic mice by targeting ADAM12, an extracellular proteolytic enzyme that plays a role in extracellular matrix remodeling and post ischemic angiogenesis. In non-diabetic mice, the expression of miR-29a is significantly decreased after HLI, whereas in diabetic mice, its expression is increased. Using bioinformatics tools, we identified ADAM12 to be one of the targets of miR-29a. The expression of ADAM12 is increased in post-HLI ischemic limbs of non-diabetic mice, which would be expected if miR-29a targets ADAM12 transcript to repress its expression. On the other hand, in diabetic mice, the post-HLI expression of ADAM12 was significantly lower than the non-diabetic mice. This is in accord with the higher expression of miR-29a in these tissues. Interestingly, inhibition of miR-29a in ischemic limbs of diabetic mice by antagomir, increased ADAM12 expression and improved the post-ischemic tissue repair and blood perfusion. Moreover, we have identified several other miRNAs in these experiments whose expression is modulated in the ischemic limbs of diabetic mice (unpublished results). These results strongly suggest that altered miRNA expression in ischemic diabetic tissues are likely potent contributors to poor PAD outcomes in diabetes. Identifying the target of miRNAs with altered expression in ischemic diabetic tissues may provide novel insights into the molecular mechanisms by with diabetes contributes to poor PAD outcomes. It will be interesting to know whether these miRNAs act in concert with one another to affect the genes involved in the same pathway or they target genes involved in additional pathways.

To this end, a combination of miRNA expression by global miRNA sequencing (miRseq) and mRNA sequencing (RNAseq) or mRNA array analyses will be highly informative in identifying differences in miRNA and their target genes that are critical in diabetes-induced pathology. Using such approach endothelial-enriched miRNAs miR-615-5p was identified to be downregulated under pro-angiogenic conditions and upregulated in wounds including in diabetic mice (db/db mice). Inhibition of miR-615-5p by synthetic antagomir improved blood perfusion and angiogenesis after surgical hind limb ischemia in diabetic db/db mice (91).

In addition to identifying the miRNAs that shape the outcomes of diabetic ischemic limb injury, it is also important to understand the regulation of expression of these miRNAs. A number of miRNAs are embedded in protein-coding genes and their expression is closely synchronized with the host genes. However, miRNAs also exist independently in the genome, and little is known of the regulation of their expression. In our studies, the miR-29a location has been known to exist independently. The information on its expression and regulatory cis-elements or transcriptional factors is not known. It will be important to understand how the expression of this miRNA is regulated under ischemic conditions and how the expression is influenced by the metabolic abnormalities in diabetes leading to altered expression of its target genes.

## Genetic mapping and role of limb salvage QTL-1 (LSQ-1) genes on diabetic PAD outcomes

Our laboratory's interest has been to understand the molecular mechanisms of recovery from ischemic limb injury in PAD, and how diabetes adversely affects the outcomes of post-ischemic recovery. Using a well-established mouse hind limb ischemia (HLI) model of PAD, we have taken a genetic mapping approach to identify genes that have major impact on post-ischemic recovery, explore their mechanism of action followed by investigations of the effect of diabetes on the expression and function of these genes. We identified a genetic locus on C57Bl/6 mouse chromosome 7 termed LSQ-1 that determines good outcomes following ischemic limb injury (92). This approach led us to map a variant *Bag3* gene that functions as a key co-chaperone in autophagy, and also an anti-apoptotic protein (93). We have shown that BAG3 may be a key determinant of post-ischemic recovery in mouse HLI. In diabetic mice (high fat diet), we observed high rates of limb loss compared to non-diabetic mice, likely due to poorer perfusion, lesser degree of angiogenesis, and a more severe post ischemic skeletal muscle injury. These findings suggest that despite the adequate post-HLI adaptation and recovery of C57BL/6 mice, high fat diet or hyperglycemia (HFD + STZ, or Akita mice) alters the expression of a key molecule involved in post-injury repair of limb tissue. Interestingly the ischemic hind limb skeletal muscles of diabetic C57BL/6J mice showed significantly lower expression of two genes mapped on Lsq-1 locus, i.e., BAG3 and ADAM12, compared to that in non-diabetic mouse limbs (64, 68). Exogenous expression of BAG3 in the limbs of diabetic mice prior to HLI significantly reduced limb loss, improved perfusion recovery and decreased extent of skeletal muscle injury (68). The repression of gene expression in diabetes could result from either transcriptional regulation or from post-transcriptional regulatory mechanisms, such as microRNAs. At least in the case of ADAM12, we have identified a regulatory mechanism whereby increased expression of miRNA-29a in post-HLI limb tissue results in decreased ADAM12 expression. In contrast, neutralization of miR-29a by exogenous antagomir prevents the loss of ADAM12 in post-HLI limbs and improves perfusion recovery and tissue damage (64). In the future studies, it will be important to understand these mechanisms that may identify transcription factors, *Bag3* promoter regulatory sequences, and specific miRNAs of potential therapeutic use. It also remains to be determined whether poor post-HLI recovery resulting from BAG3 deficiency is cell type specific. Genetically modified mice to either knockout *Bag3* in cell type-specific manner or to selectively express BAG3 protein in specific cell types is likely to be very informative.

## Conclusion

While it is well established that diabetes contributes to the development and progression of arteriosclerosis, the primary cause of vessel occlusion in PAD, the effect of diabetes on PAD goes beyond development of the disease. Diabetes is a major driver of disease severity likely through its effect on cellular and molecular processes involved in vascular and skeletal muscle adaption to ischemia. The precise mechanism of how diabetes has this profound effect on PAD outcomes is poorly understood and remain an area of great need for investigation.
